# Supplementary material for: ProteinShader: illustrative rendering of macromolecules
Source: BMC Struct Biol. 2009 Mar 30;9:19. doi: 10.1186/1472-6807-9-19 (PMC2672931; doi:10.1186/1472-6807-9-19)
Supplement: Additional file 1 — ProteinShader program without source code. This compressed file contains the complete ProteinShader program including associated libraries, but no source code. A README.txt file gives an overview of the ProteinShader distribution, and the index.html file in the help subdirectory has directions on getting started with the program as well as a set of tutorials. [file 1472-6807-9-19-S1.zip › ProteinShader-beta-0_9_4-binary/help/api/org/proteinshader/gui/components/controlpanel/AtomColorPanel.html]

AtomColorPanel (ProteinShader API)


|  |  |  |
| --- | --- | --- |
| **Overview** \| **Package** \| **Class** \| **Use** \| **Tree** \| **Deprecated** \| **Index** \| **Help** | |  |
| **PREV CLASS**   **NEXT CLASS** | **FRAMES**    **NO FRAMES**     **All Classes** |  |
| SUMMARY: NESTED \| FIELD \| CONSTR \| METHOD | DETAIL: FIELD \| CONSTR \| METHOD |  |


---


## org.proteinshader.gui.components.controlpanel Class AtomColorPanel

```
java.lang.Object
  java.awt.Component
      java.awt.Container
          javax.swing.JComponent
              javax.swing.JPanel
                  org.proteinshader.gui.components.controlpanel.ColorPanel
                      org.proteinshader.gui.components.controlpanel.AtomColorPanel
```

**All Implemented Interfaces:**: ImageObserver, MenuContainer, Serializable, Accessible

---

``` public class AtomColorPanel extends ColorPanel ```

This control panel allows the user to modify the color of Atoms.

**See Also:**: Serialized Form

---

**Nested Class Summary**

**Nested classes/interfaces inherited from class javax.swing.JPanel**

`JPanel.AccessibleJPanel`

**Nested classes/interfaces inherited from class javax.swing.JComponent**

`JComponent.AccessibleJComponent`

**Nested classes/interfaces inherited from class java.awt.Container**

`Container.AccessibleAWTContainer`

**Nested classes/interfaces inherited from class java.awt.Component**

`Component.AccessibleAWTComponent, Component.BltBufferStrategy, Component.FlipBufferStrategy`


| **Field Summary** | |
| --- | --- |
| `static String` | `MENU_NAME`             The menu name "Color" will be returned by toString(). |

**Fields inherited from class javax.swing.JComponent**

`accessibleContext, listenerList, TOOL_TIP_TEXT_KEY, ui, UNDEFINED_CONDITION, WHEN_ANCESTOR_OF_FOCUSED_COMPONENT, WHEN_FOCUSED, WHEN_IN_FOCUSED_WINDOW`

**Fields inherited from class java.awt.Component**

`BOTTOM_ALIGNMENT, CENTER_ALIGNMENT, LEFT_ALIGNMENT, RIGHT_ALIGNMENT, TOP_ALIGNMENT`

**Fields inherited from interface java.awt.image.ImageObserver**

`ABORT, ALLBITS, ERROR, FRAMEBITS, HEIGHT, PROPERTIES, SOMEBITS, WIDTH`


**Constructor Summary**

`AtomColorPanel(Mediator mediator,
Frame dialogOwner,
RadioPanel radioPanel)`
  
          Constructs a ColorPanel.


| **Method Summary** | |
| --- | --- |
| `void` | `modifyGlobal(float red, float green, float blue)`             Prepares the AtomModifier to modify the RGB color of Atoms, and then calls on the modifyGlobal() method of the RadioPanel. |
| `void` | `modifyGlobalToDefault1()`             Default 1 sets the RGB color of Atoms based on Atom type. |
| `void` | `modifyGlobalToDefault2()`             Default 2 sets the RGB color of Atoms based on AminoAcid type. |
| `void` | `modifyHelices(float red, float green, float blue)`             Prepares the the AtomModifier to modify the RGB color of Atoms, and then calls on the modifyHelices() method of the RadioPanel. |
| `void` | `modifyHelicesToDefault1()`             Default 1 sets the RGB color of Atoms based on Atom type. |
| `void` | `modifyHelicesToDefault2()`             Default 2 sets the RGB color of Atoms based on AminoAcid type. |
| `void` | `modifyLoops(float red, float green, float blue)`             Prepares the AtomModifier to modify the RGB color of Atoms, and then calls on the modifyLoops() method of the RadioPanel. |
| `void` | `modifyLoopsToDefault1()`             Default 1 sets the RGB color of Atoms based on Atom type. |
| `void` | `modifyLoopsToDefault2()`             Default 2 sets the RGB color of Atoms based on AminoAcid type. |
| `void` | `modifySelected(float red, float green, float blue)`             Prepares the AtomModifier to modify the RGB color of Atoms, and then calls on the modifySelected() method of the RadioPanel. |
| `void` | `modifySelectedToDefault1()`             Default 1 sets the RGB color of Atoms based on Atom type. |
| `void` | `modifySelectedToDefault2()`             Default 2 sets the RGB color of Atoms based on AminoAcid type. |
| `void` | `modifyStrands(float red, float green, float blue)`             Prepares the AtomModifier to modify the RGB color of Atoms, and then calls on the modifyStrands() method of the RadioPanel. |
| `void` | `modifyStrandsToDefault1()`             Default 1 sets the RGB color of Atoms based on Atom type. |
| `void` | `modifyStrandsToDefault2()`             Default 2 sets the RGB color of Atoms based on AminoAcid type. |
| `void` | `setColor(float red, float green, float blue)`             Clears the Atom modifier and sets the RGB colors. |
| `String` | `toString()`             Returns a name suitable for use in a menu. |

**Methods inherited from class org.proteinshader.gui.components.controlpanel.ColorPanel**

`applyColors, applyDefaultColors1, applyDefaultColors2, modifyGlobal, modifyHelices, modifyLoops, modifySelected, modifyStrands, openColorChooser`

**Methods inherited from class javax.swing.JPanel**

`getAccessibleContext, getUI, getUIClassID, paramString, setUI, updateUI`

**Methods inherited from class javax.swing.JComponent**

`addAncestorListener, addNotify, addVetoableChangeListener, computeVisibleRect, contains, createToolTip, disable, enable, firePropertyChange, firePropertyChange, firePropertyChange, fireVetoableChange, getActionForKeyStroke, getActionMap, getAlignmentX, getAlignmentY, getAncestorListeners, getAutoscrolls, getBorder, getBounds, getClientProperty, getComponentGraphics, getComponentPopupMenu, getConditionForKeyStroke, getDebugGraphicsOptions, getDefaultLocale, getFontMetrics, getGraphics, getHeight, getInheritsPopupMenu, getInputMap, getInputMap, getInputVerifier, getInsets, getInsets, getListeners, getLocation, getMaximumSize, getMinimumSize, getNextFocusableComponent, getPopupLocation, getPreferredSize, getRegisteredKeyStrokes, getRootPane, getSize, getToolTipLocation, getToolTipText, getToolTipText, getTopLevelAncestor, getTransferHandler, getVerifyInputWhenFocusTarget, getVetoableChangeListeners, getVisibleRect, getWidth, getX, getY, grabFocus, isDoubleBuffered, isLightweightComponent, isManagingFocus, isOpaque, isOptimizedDrawingEnabled, isPaintingTile, isRequestFocusEnabled, isValidateRoot, paint, paintBorder, paintChildren, paintComponent, paintImmediately, paintImmediately, print, printAll, printBorder, printChildren, printComponent, processComponentKeyEvent, processKeyBinding, processKeyEvent, processMouseEvent, processMouseMotionEvent, putClientProperty, registerKeyboardAction, registerKeyboardAction, removeAncestorListener, removeNotify, removeVetoableChangeListener, repaint, repaint, requestDefaultFocus, requestFocus, requestFocus, requestFocusInWindow, requestFocusInWindow, resetKeyboardActions, reshape, revalidate, scrollRectToVisible, setActionMap, setAlignmentX, setAlignmentY, setAutoscrolls, setBackground, setBorder, setComponentPopupMenu, setDebugGraphicsOptions, setDefaultLocale, setDoubleBuffered, setEnabled, setFocusTraversalKeys, setFont, setForeground, setInheritsPopupMenu, setInputMap, setInputVerifier, setMaximumSize, setMinimumSize, setNextFocusableComponent, setOpaque, setPreferredSize, setRequestFocusEnabled, setToolTipText, setTransferHandler, setUI, setVerifyInputWhenFocusTarget, setVisible, unregisterKeyboardAction, update`

**Methods inherited from class java.awt.Container**

`add, add, add, add, add, addContainerListener, addImpl, addPropertyChangeListener, addPropertyChangeListener, applyComponentOrientation, areFocusTraversalKeysSet, countComponents, deliverEvent, doLayout, findComponentAt, findComponentAt, getComponent, getComponentAt, getComponentAt, getComponentCount, getComponents, getComponentZOrder, getContainerListeners, getFocusTraversalKeys, getFocusTraversalPolicy, getLayout, getMousePosition, insets, invalidate, isAncestorOf, isFocusCycleRoot, isFocusCycleRoot, isFocusTraversalPolicyProvider, isFocusTraversalPolicySet, layout, list, list, locate, minimumSize, paintComponents, preferredSize, printComponents, processContainerEvent, processEvent, remove, remove, removeAll, removeContainerListener, setComponentZOrder, setFocusCycleRoot, setFocusTraversalPolicy, setFocusTraversalPolicyProvider, setLayout, transferFocusBackward, transferFocusDownCycle, validate, validateTree`

**Methods inherited from class java.awt.Component**

`action, add, addComponentListener, addFocusListener, addHierarchyBoundsListener, addHierarchyListener, addInputMethodListener, addKeyListener, addMouseListener, addMouseMotionListener, addMouseWheelListener, bounds, checkImage, checkImage, coalesceEvents, contains, createImage, createImage, createVolatileImage, createVolatileImage, disableEvents, dispatchEvent, enable, enableEvents, enableInputMethods, firePropertyChange, firePropertyChange, firePropertyChange, firePropertyChange, firePropertyChange, firePropertyChange, getBackground, getBounds, getColorModel, getComponentListeners, getComponentOrientation, getCursor, getDropTarget, getFocusCycleRootAncestor, getFocusListeners, getFocusTraversalKeysEnabled, getFont, getForeground, getGraphicsConfiguration, getHierarchyBoundsListeners, getHierarchyListeners, getIgnoreRepaint, getInputContext, getInputMethodListeners, getInputMethodRequests, getKeyListeners, getLocale, getLocation, getLocationOnScreen, getMouseListeners, getMouseMotionListeners, getMousePosition, getMouseWheelListeners, getName, getParent, getPeer, getPropertyChangeListeners, getPropertyChangeListeners, getSize, getToolkit, getTreeLock, gotFocus, handleEvent, hasFocus, hide, imageUpdate, inside, isBackgroundSet, isCursorSet, isDisplayable, isEnabled, isFocusable, isFocusOwner, isFocusTraversable, isFontSet, isForegroundSet, isLightweight, isMaximumSizeSet, isMinimumSizeSet, isPreferredSizeSet, isShowing, isValid, isVisible, keyDown, keyUp, list, list, list, location, lostFocus, mouseDown, mouseDrag, mouseEnter, mouseExit, mouseMove, mouseUp, move, nextFocus, paintAll, postEvent, prepareImage, prepareImage, processComponentEvent, processFocusEvent, processHierarchyBoundsEvent, processHierarchyEvent, processInputMethodEvent, processMouseWheelEvent, remove, removeComponentListener, removeFocusListener, removeHierarchyBoundsListener, removeHierarchyListener, removeInputMethodListener, removeKeyListener, removeMouseListener, removeMouseMotionListener, removeMouseWheelListener, removePropertyChangeListener, removePropertyChangeListener, repaint, repaint, repaint, resize, resize, setBounds, setBounds, setComponentOrientation, setCursor, setDropTarget, setFocusable, setFocusTraversalKeysEnabled, setIgnoreRepaint, setLocale, setLocation, setLocation, setName, setSize, setSize, show, show, size, transferFocus, transferFocusUpCycle`

**Methods inherited from class java.lang.Object**

`clone, equals, finalize, getClass, hashCode, notify, notifyAll, wait, wait, wait`

**Field Detail**

### MENU\_NAME

```
public static final String MENU_NAME
```

:   The menu name "Color" will be returned by toString().

    **See Also:**: Constant Field Values


**Constructor Detail**

### AtomColorPanel

```
public AtomColorPanel(Mediator mediator,
                      Frame dialogOwner,
                      RadioPanel radioPanel)
```

:   Constructs a ColorPanel.

    **Parameters:**: `mediator` - the centralized Mediator that most listeners need to call on to accomplish their task.: `dialogOwner` - the owner of any Dialogs opened from the SelectorPanel (or null if there is no requested owner).: `radioPanel` - the common RadioPanel used by subpanels.


**Method Detail**

### modifySelected

```
public void modifySelected(float red,
                           float green,
                           float blue)
                    throws ColorOutOfRangeException
```

:   Prepares the AtomModifier to modify the RGB color of Atoms, and
    then calls on the modifySelected() method of the RadioPanel.

    :   **Specified by:**: `modifySelected` in class `ColorPanel`
    :   **Parameters:**: `red` - the red component of the RGB color to set.: `green` - the green component of the RGB color to set.: `blue` - the blue component of the RGB color to set. **Throws:**: `ColorOutOfRangeException` - if a color is less than 0.0 or greater than 1.0.

---


### modifyHelices

```
public void modifyHelices(float red,
                          float green,
                          float blue)
                   throws ColorOutOfRangeException
```

:   Prepares the the AtomModifier to modify the RGB color of Atoms,
    and then calls on the modifyHelices() method of the RadioPanel.

    :   **Specified by:**: `modifyHelices` in class `ColorPanel`
    :   **Parameters:**: `red` - the red component of the RGB color to set.: `green` - the green component of the RGB color to set.: `blue` - the blue component of the RGB color to set. **Throws:**: `ColorOutOfRangeException` - if a color is less than 0.0 or greater than 1.0.

---


### modifyStrands

```
public void modifyStrands(float red,
                          float green,
                          float blue)
                   throws ColorOutOfRangeException
```

:   Prepares the AtomModifier to modify the RGB color of Atoms,
    and then calls on the modifyStrands() method of the RadioPanel.

    :   **Specified by:**: `modifyStrands` in class `ColorPanel`
    :   **Parameters:**: `red` - the red component of the RGB color to set.: `green` - the green component of the RGB color to set.: `blue` - the blue component of the RGB color to set. **Throws:**: `ColorOutOfRangeException` - if a color is less than 0.0 or greater than 1.0.

---


### modifyLoops

```
public void modifyLoops(float red,
                        float green,
                        float blue)
                 throws ColorOutOfRangeException
```

:   Prepares the AtomModifier to modify the RGB color of Atoms,
    and then calls on the modifyLoops() method of the RadioPanel.

    :   **Specified by:**: `modifyLoops` in class `ColorPanel`
    :   **Parameters:**: `red` - the red component of the RGB color to set.: `green` - the green component of the RGB color to set.: `blue` - the blue component of the RGB color to set. **Throws:**: `ColorOutOfRangeException` - if a color is less than 0.0 or greater than 1.0.

---


### modifyGlobal

```
public void modifyGlobal(float red,
                         float green,
                         float blue)
                  throws ColorOutOfRangeException
```

:   Prepares the AtomModifier to modify the RGB color of Atoms, and
    then calls on the modifyGlobal() method of the RadioPanel.

    :   **Specified by:**: `modifyGlobal` in class `ColorPanel`
    :   **Parameters:**: `red` - the red component of the RGB color to set.: `green` - the green component of the RGB color to set.: `blue` - the blue component of the RGB color to set. **Throws:**: `ColorOutOfRangeException` - if a color is less than 0.0 or greater than 1.0.

---


### modifySelectedToDefault1

```
public void modifySelectedToDefault1()
```

:   Default 1 sets the RGB color of Atoms based on Atom type.

---


### modifyHelicesToDefault1

```
public void modifyHelicesToDefault1()
```

:   Default 1 sets the RGB color of Atoms based on Atom type.

---


### modifyStrandsToDefault1

```
public void modifyStrandsToDefault1()
```

:   Default 1 sets the RGB color of Atoms based on Atom type.

---


### modifyLoopsToDefault1

```
public void modifyLoopsToDefault1()
```

:   Default 1 sets the RGB color of Atoms based on Atom type.

---


### modifyGlobalToDefault1

```
public void modifyGlobalToDefault1()
```

:   Default 1 sets the RGB color of Atoms based on Atom type.

---


### modifySelectedToDefault2

```
public void modifySelectedToDefault2()
```

:   Default 2 sets the RGB color of Atoms based on AminoAcid type.
    There is no effect if there the Atom does not belong to an
    AminoAcid.

---


### modifyHelicesToDefault2

```
public void modifyHelicesToDefault2()
```

:   Default 2 sets the RGB color of Atoms based on AminoAcid type.
    There is no effect if there the Atom does not belong to an
    AminoAcid.

---


### modifyStrandsToDefault2

```
public void modifyStrandsToDefault2()
```

:   Default 2 sets the RGB color of Atoms based on AminoAcid type.
    There is no effect if there the Atom does not belong to an
    AminoAcid.

---


### modifyLoopsToDefault2

```
public void modifyLoopsToDefault2()
```

:   Default 2 sets the RGB color of Atoms based on AminoAcid type.
    There is no effect if there the Atom does not belong to an
    AminoAcid.

---


### modifyGlobalToDefault2

```
public void modifyGlobalToDefault2()
```

:   Default 2 sets the RGB color of Atoms based on AminoAcid type.
    There is no effect if there the Atom does not belong to an
    AminoAcid.

---


### toString

```
public String toString()
```

:   Returns a name suitable for use in a menu.

    :   **Overrides:**: `toString` in class `Component`
    :   **Returns:**: The menu name as a String.

---


### setColor

```
public void setColor(float red,
                     float green,
                     float blue)
              throws ColorOutOfRangeException
```

:   Clears the Atom modifier and sets the RGB colors.

    :   **Parameters:**: `red` - the red component of the RGB color to set.: `green` - the green component of the RGB color to set.: `blue` - the blue component of the RGB color to set. **Throws:**: `ColorOutOfRangeException` - if a color is less than 0.0 or greater than 1.0.


---


|  |  |  |
| --- | --- | --- |
| **Overview** \| **Package** \| **Class** \| **Use** \| **Tree** \| **Deprecated** \| **Index** \| **Help** | |  |
| **PREV CLASS**   **NEXT CLASS** | **FRAMES**    **NO FRAMES**     **All Classes** |  |
| SUMMARY: NESTED \| FIELD \| CONSTR \| METHOD | DETAIL: FIELD \| CONSTR \| METHOD |  |


---

# *Copyright © 2007-2008*
